# Molecular Dynamics Simulations Provide Further Insights into the Allosteric Regulation of the Kinesin‑5 Motor Domain by Loop 5

**DOI:** 10.1021/acs.jcim.5c02999

**Published:** 2026-03-12

**Authors:** Gabriel Rodríguez-Santos, Giorgio Bonollo, Cristiano Sciva, Giorgio Colombo, Concepción Pérez-Melero, Stefano A. Serapian

**Affiliations:** † Pharmaceutical Sciences Department, Pharmaceutical Chemistry Unit, University of Salamanca, Biomedical Research Institute of Salamanca (IBSAL), C/Licenciado Méndez Nieto s/n, Salamanca 37007, Spain; ‡ Chemistry Department, 19001 University of Pavia, via Torquato Taramelli 12, Pavia 27100, Italy; § Tropical Diseases Research Centre, University of Salamanca (CIETUS), C/Licenciado Méndez Nieto s/n, 37007 Salamanca, Spain

## Abstract

Human kinesin-5 is a protein that oversees the proper formation of the bipolar mitotic spindle and is thus an appealing target for cancer treatment. The main group of kinesin-5 inhibitors reported to date binds to an allosteric pocket formed by loop 5 (L5), which is a key structural element believed to allosterically modulate kinesin-5 functionality. In this study, we carried out extensive molecular dynamics (MD) simulations on the motor domain of kinesin-5 in four representative catalytic states: ATP-bound, ADP-bound, without nucleotide (apo), and dually bound by ADP and the known main group inhibitor filanesib. MD trajectories were analyzed using the *Distance Fluctuation* and *Shortest Path Map* methods to compare and contrast allosteric connections across different parts of the motor domain in each of the four states. Simulations show that L5 is allosterically connected to both the nucleotide-binding site and the kinesin-5–microtubule interface. In the absence of inhibitor, L5 alternates between a “docked” conformation in the ATP and apo states and an “undocked” conformation in the ADP state. This supports the idea that the L5 binding pocket is cryptic and that inhibitor binding takes place in the ADP state. Residues Trp127 and Tyr211 were found to be crucial for the L5 conformational alternation. Once filanesib binds to the ADP form, we found that L5 stabilizes into an ATP-like conformation that prevents ADP release, possibly via sequestration of Glu118 by filanesib itself. Additionally, the presence of filanesib intensifies anomalous allosteric connections with L8, which is a crucial mediator of microtubule binding. This could explain the low affinity of kinesin-5 for the microtubule when L5 inhibitors are present. Our findings allow a deeper understanding of the key role of L5 in regulating kinesin-5 activity and how L5 inhibitors can achieve its disruption.

## Graphical abstract

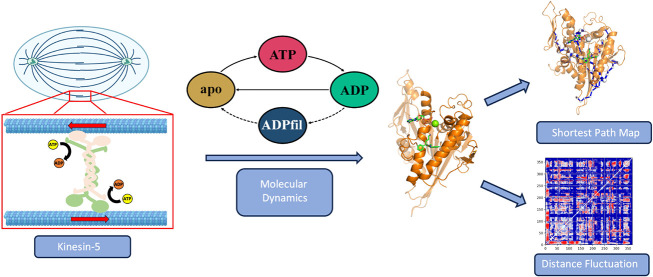

## 1 Introduction

Cancer is one of the most prevalent diseases in the world, and its incidence is expected to increase drastically over the next 25 years. Chemotherapy is the main strategy for treating malignancies, but chemotherapeutic agents have to deal with their intrinsic toxicity, which is often related to their own mechanism of action; this has a direct consequence on their adverse reaction profile, which often limits their dosing. For instance, the antimitotic agents currently used in clinic (taxanes, vinca alkaloids, and epothilones) target the human tubulin, altering its normal dynamics. Since tubulin is an essential component of the mitotic spindle, its inhibition causes mitotic arrest and cell death; but, it is also required for the function of healthy cells, which is the main reason for antimitotic neurotoxicity that limits their dosage.^1–3^ The mitotic kinesin-5, a protein required for the development of the bipolar mitotic spindle and the proper placement of chromosomes on the metaphase plate, appears to be an attractive target for the development of new antimitotic drugs,^4^ as its role in tumor onset and progression is well established, being overexpressed in different tumoral cells.^5–10^

Structurally, kinesin-5 is a homotetramer organized into two antiparallel units (dumbbell shape; Figure 1A), each one bound to a microtubule originating from each pole of the cell in such a way that the microtubules are cross-linked. Each monomer is composed of an N-terminal motor domain, a neck linker, a coiled stalk, and a tail domain.^11,12^ The motor domain is responsible for ATP hydrolysis and microtubule binding; the neck linker modulates the coordinated movement of every motor domain within each pair; and the coiled domain is responsible for the aggregation of all four monomers, first by interlacing two monomers to form a dimer, which further coils with another dimer. In addition, the coiled domain is thought to interact with the opposing dimer by slowing its movement and, by doing so, increasing the coordination of the whole tetramer.^13^ Through ATP hydrolysis, the kinesin, which is directed to the (+)-end of the microtubule, generates the pushing force that is necessary for bipolar spindle generation, cell elongation, and the proper placement of chromosomes on the mitotic spindle. Its inhibition causes the cell to exit mitosis too prematurely and, therefore, provokes cell death due to either apoptosis or the generation of aberrant cells (Figure 1B). Additionally, it has been demonstrated that it has a stabilizing effect on growing microtubules, which is needed for the development of the spindle.^14^

**Figure 1:**
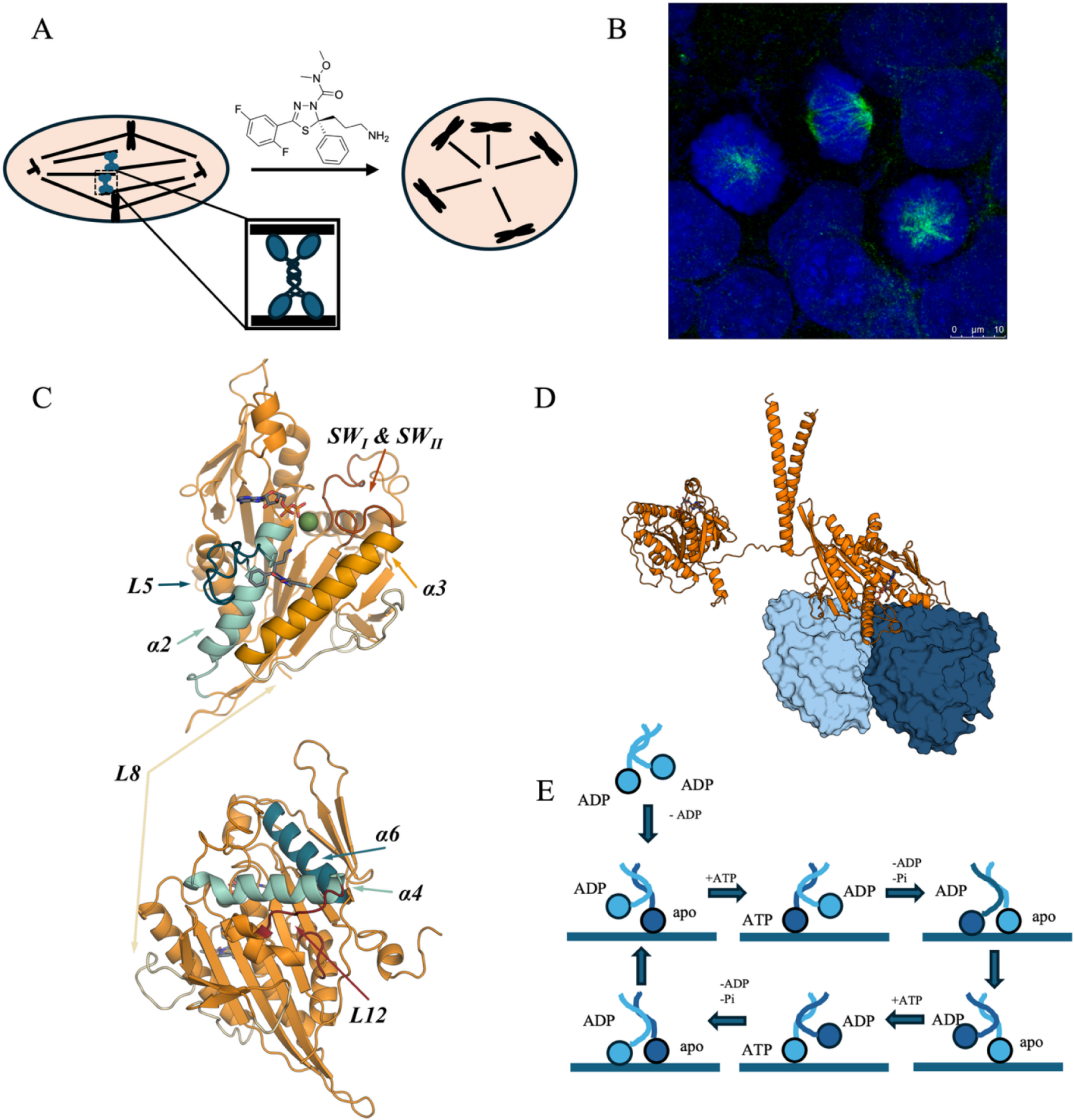
Kinesin-5 is necessary for mitotic spindle development. A) Schematic representation of filanesib inhibition of kinesin-5 in a mitotic cell, producing a monopolar spindle. B) Fluorescence microscopy image of a normal bipolar mitotic spindle (middle) and two monopolar spindles due to kinesin-5 inhibition.^58^ C) Kinesin-5 motor domain. Key regions (including L5) are labeled and highlighted in different colors; remaining regions are rendered in dark orange. D) Model of a kinesin-5 dimer (both protomers in orange) with one of the motor domains bound to an αβ-tubulin dimer (cyan and blue), using the Cryo-EM structure of kinesin-5 with AMPNP (PDB ID: 6TA4) as a template. From the model, it can be seen that the binding interface of kinesin-5 with the tubulin dimer is composed of L2, L8, SWII, α4, L12, and α6.^59^ E) Schematic representation of the catalytic cycle of a kinesin-5 dimer. B) was reproduced from ref 58 with permission of the Royal Society of Chemistry.

The motor domain of kinesin-5 (Figure 1C) shares some common features with the motor domain of other members of the kinesin superfamily, i.e., a core of 8 β-sheets surrounded by 6 α-helices.^12^ The catalytic domain is composed of a P-loop and two switch domains (SWI and SWII), whose key residues—Ser233, Arg234, and Glu264 (the last two forming a salt bridge)—are sensitive to the presence of ATP γ-phosphate and responsible for its hydrolysis in all kinesins.^15^ The switch domains play a role similar to that in G-proteins and in myosin, and they transmit the effect of nucleotide hydrolysis to other key regions through allosteric routes. Some of these regions are the α4 helix, which together with L12 and L8 forms the interface with the microtubule (MT; Figure 1D), and α6, which is directly connected to the neck linker.

Like other kinesins, kinesin-5 has a loop (L5, Glu116-Gly134) that protrudes from α2. Interestingly, L5 of kinesin-5 is the longest among the superfamily of kinesins.^16^ It has been proposed that this and other small structural differences cause the fine-tuning of the kinetic parameters of kinesins, adapting them so they best fit the task they are designed for.^17,18^ For instance, kinesin-1 is perfectly adapted to the transport of vesicles, which needs to be quick and does not require a strong force. The exact opposite occurs with the assembly of the mitotic spindle that is carried out by kinesin-5, which does not have a requirement for high speed but undoubtedly needs to withstand strong forces in the overlapping microtubules, which, in extreme situations, would lock kinesin-5 in a “handbrake-state”.^19–21^ This is further supported by the fact that a chimeric kinesin-5 of *Xenopus laevis*, in which the motor domains were replaced by those of kinesin-1, could glide along two antiparallel microtubules but failed to assemble into a proper bipolar spindle.^22^ A most dramatic example of structural differences among kinesins is provided by kinesin-3 (KIF14), which displays an *N*-terminal fragment with 350 residues (almost as much as the rest of the motor domain) believed to confer a superprocessive movement and a more efficient performance in crowded environments.^23^

L5 is essential for the kinesin-5 role, as evidenced by mutagenesis studies or by the existence of a whole class of kinesin-5 inhibitors that bind to a pocket formed by L5 itself, α2, and α3. This pocket is only present in the crystallographic structures of kinesin-5 in complex with any of these inhibitors and not in ATP- or ADP-bound representative structures, so it is believed to be induced by the presence of these inhibitors. The participation of the loop in ATP hydrolysis is clear, since kinesin mutants with a deletion of the loop display significantly lower activity. It has also been shown that L5 contributes to ADP release and prevents the motor domain from binding to the MT prior to the release of ADP.^24–27^ Also, the presence of an allosteric inhibitor does not seem to disrupt the crystallographic structure of the kinesin, contrary to what one might expect from such an allosteric inhibitor. Even more paradoxical is that some of the mutations (Asp130Val, Ala133Asp, and the double mutation) that confer resistance to inhibitors increase the enthalpic contribution to the binding free energy and only decrease the latter because of a higher entropic penalty.^28^ As mentioned before, L5 is present in all kinesins; however, human kinesin-5 inhibitors are specific to this protein, and they do not display any activity on other kinesins, even the equivalent kinesin-5 proteins of other species. Interestingly enough, chimeric nonhuman kinesins in which the human L5 replaces the wild-type L5 can be inhibited by human kinesin-5 inhibitors, which suggests that the allosteric mechanisms governed by L5 are conserved, regardless of L5 length.^29^ Nevertheless, the detailed mechanism by which L5 acts remains unclear.

The catalytic cycle that takes place in the motor domain is directly linked to the functionality of the protein, and it can be classified into three different stages: ATP-bound, ADP-bound, and apo (without ATP or ADP bound). The ATP and apo forms have a high affinity for the MT, whereas the ADP form does not; the proper succession of stages grants the processivity of the protein. In a way, the movement of the kinesin can be seen as “little man walking” (Figure 1E), with one motor head (the man’s foot) that must remain bound to the MT while the rear head (the other foot) is transposed forward, at which point it regains affinity for the MT. Once the more advanced head is reattached to the MT, the new rear head can get loose from the MT to continue the walk. First, the kinesin with both motor domains bound to ADP approaches the MT^30^ and the advanced motor domain releases ADP and tightly binds to the MT. The binding of ATP causes a conformational perturbation that is believed to be transmitted up to the neck linker, which in turn triggers a whip movement that propels the rear ADP motor domain.^15,31^ Recent works have shown that for kinesin-1, the model that best fits the experimental data is a “Brownian ratchet” in which the neck linker plays an assisting role.^32^ This model is also plausible for kinesin-5.^33^ The now advanced ADP motor domain releases the ADP^34^ and attaches to the MT in a transition to a high-microtubule-affinity apo form,^17^ allowing the ATP motor domain left behind to hydrolyze ATP and get loose from the MT. The recently acquired apo state induces a conformational change in tubulin that enhances binding affinity,^35,36^ and the binding of a new ATP molecule to the first head restarts the cycle. In stepping motors, this is translated into motility, while in static motors (as is the case of kinesin-5), this force is channeled through the MT to which is bound,^16^ and as both ends of the kinesin are bound to antiparallel MTs, they will slide apart. It is the alternation of the stepping and braking effects of the kinesin that enables spindle formation.^16^

To date, there are two known types of kinesin-5 inhibitors, both allosteric and known to act by two essentially opposing mechanisms, which are based, in any case, on disrupting this catalytic cycle. The main group of inhibitors binds to the previously mentioned pocket formed by L5, α2, and α3 and is reported to stabilize an “ADP-like” state characterized by a decreased release of ADP and a low affinity for MT, as manifested by crystallographic and kinetic data.^37,38^ The same kinetic experiments show that these inhibitors do not disrupt the binding of ATP, that is, the transition from apo to ATP-bound, meaning that their binding must take place at either the ATP or ADP form. This is further supported by the calorimetric experiments in the presence of the inhibitor ispinesib performed by Sheth and coworkers,^39^ who also showed that the binding of the inhibitors cannot take place in the apo form. In any case, the kinetic experiments that demonstrate that these inhibitors slow ADP release,^24,37^ and the large data set of L5 inhibitors in complex with kinesin-5 show that the inhibitors remain bound to the motor domain in an ADP state. Some members of this group of inhibitors have reached phase I/II clinical trials, such as ispinesib,^40^ AZD4877,^41^ SB743921,^42^ litronesib,^43^ and filanesib,^44^ showing promising results, especially in combination with other drugs, such as proteasome inhibitors and dexamethasone. However, none of them have yet reached the clinic. Among these, filanesib is particularly amenable to study in light of its greater conformational stability and longer half-life (>90 h compared to <30 h).^12^

Coupled with suitable analyses, molecular dynamics (MD) simulations represent an ideal tool to elucidate differential allosteric effects in the various stages of the ATPase cycle in kinesin-5 motor domains (and in the presence of an allosteric inhibitor). Although there are a number of molecular dynamics publications on the motor domain of kinesin-5,^45–57^ only a minority of them explicitly address the allosteric phenomena that govern kinesin-5 inhibition by L5 ligands.

The study we present here, on the other hand, employs extensive MD simulations to elucidate and compare, in detail, all the allosteric communications occurring throughout the motor domain at key stages of the catalytic cycle. In the cycle, we have also included the apo form, with a special emphasis on the role of L5, providing significant new insights into the allosteric mechanism through which filanesib interferes with the functional cycle of kinesin-5. Such a systematic comparison of these four forms through a microsecond-long MD simulation is something that has not been attempted thus far.

## 2 Materials and Methods

We conducted molecular dynamics simulations of 4 representative states of the catalytic cycle of the motor domain of kinesin-5. These states are the ATP-bound (**ATP**), ADP-bound (**ADP**), and apo (**apo**; neither nucleotide nor inhibitor), with a fourth state (the ADP form in complex with filanesib; henceforth, **ADPfil**) treated as a deviation from the normal catalytic cycle (Figure 2A). Each form was subjected to 6 independent MD replicas, each 1 μs in length; we then ran a number of analyses on the combined MD trajectories to assess allosteric correlation.

**Figure 2:**
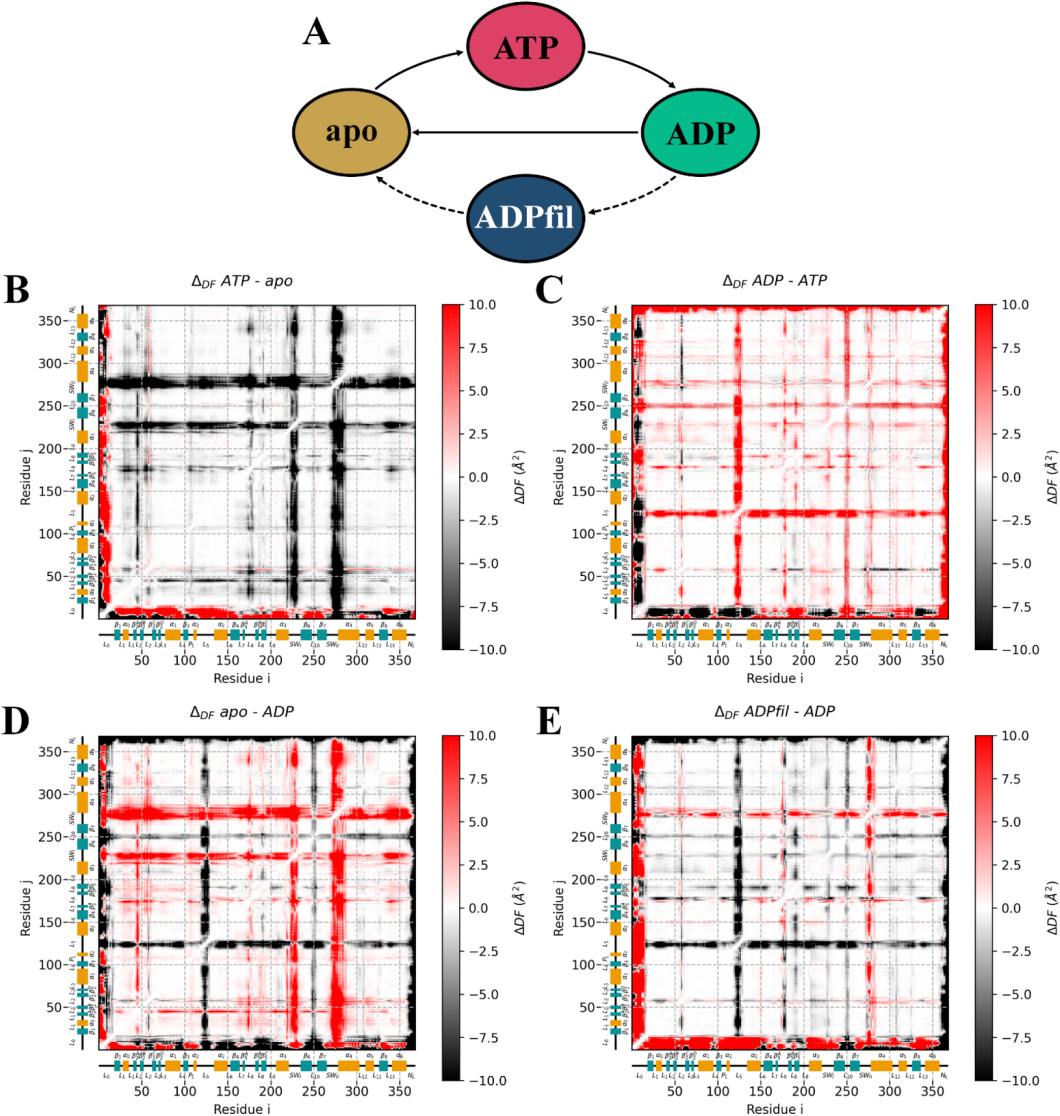
A) Schematic representation of the catalytic cycle for the kinesin-5 motor domain. B-–E) Δ*DF* matrices for the catalytic steps of kinesin-5. The Δ*DF* values were calculated as DF scores of the end point minus DF scores of the starting point, following the order of the catalytic cycle. For each state, DF values are obtained as an average of the 6 independent MD replicas combined. All of the axes contain a representation of the secondary structure of the motor domain of kinesin-5.

### 2.1 Protein Preparation

**ADP** and **ADPfil** were prepared from their respective high-resolution crystal structures (PDB IDs: 1II6 ^60^ and 6HKY,^61^ respectively). The crystal structure 6HKY features three monomers with minimal structural differences (RMSD: B to A: 0.5 Å; C to A: 3.3 Å); we ultimately chose chain A, as it had the highest number of resolved residues.

The ATP form was prepared from the crystal structure of the protein bound to the nonhydrolyzable analogue AMPPNP (PDB ID: 3HQD^62^) by replacing the N atom of the imidodiphosphate group with an oxygen.

At the time of writing, there was no reported structure for the human apo form, so we decided to model it from the human ATP form by removing the nucleotide and using the crystal structure of the apo OSM-3 kinesin from *Caenorhabditis elegans* (PDB ID: 7A40) as an additional template. The OSM-3 kinesin and human kinesin-5 share high structural similarity around the ATP binding pocket^63^ and overall: since the backbones of the apo and AMPPNP-bound forms of OSM-3 (PDB ID: 7A5E) deviate by merely 1.4 Å, and in turn, AMPPNP-bound OSM-3 deviates from AMPPNP-bound human kinesin-5 by 2.4 Å, we inferred that apo human kinesin-5 should be similar to the apo form of OSM-3. Another reason for choosing **ATP** as the starting structure for **apo** is that we reasoned both the ATP-bound and apo forms present high affinity for the microtubule and therefore must present an overall similar conformation. This is further supported by the fact that kinesin-5 inhibitors that bind to the α4 and α6 site and stabilize the binding with the microtubule induce more conformational similarity to the ATP form compared to the ADP form.^59^

Although the sequences of the two AMPPNP-bound kinesins are different, the overall structures of apo OSM-3 and AMPPNP-bound kinesin-5 are, as stated earlier, highly similar. The only differences are L5 (which is far shorter in the *C. elegans* form) and the α3 helix, which in the *C. elegans* form shows a small tilt. It should be noted that the C-terminus of helix α3 in kinesins directly connects to the SWI domain. We therefore decided to model the section corresponding to the residues 192–205 (end of α3 helix and SWI) from the *C. elegans* kinesin into the homologous region of the human kinesin (residues 221–234). It is also worth mentioning that the SWI domain from OSM-3 also contains the catalytically relevant sequence SSRSHS (residues 232–237 in human kinesin). The remaining residues in that segment were point-mutated, respecting side-chain orientation, so that they matched the human kinesin sequence. All remaining **apo** residues (including L5) were directly inherited from **ATP**.

As is customary, missing residues in all forms were reconstructed using Modeller (v. 10.5),^64^ based on the protein sequence available at Uniprot (ID P52732). This included residues 1–16 in all structures, except for **ADP** (residues 1–15). Input scripts for Modeller are provided electronically. The *reduce* utility from Antechamber was used to add the hydrogen atoms, suggest likely histidine tautomers, and model the optimal orientations of Gln/Asn side chains. The selection of the most probable protonation states of the residues at physiological pH was performed using PropKa 3.1.^65^ ATP, ADP, and filanesib hydrogens were added with *tleap*. The systems were solvated with 12 Å^3^ cubical boxes of TIP3P water, and NaCl was added to neutralize the system and to reach an ionic strength of 0.1 M using *tleap*.^66^

### 2.2 Ligand Parametrization and Force Field Selection

Parameters for ATP and ADP were obtained from the AMBER parameter database.^67^ Mg^2+^ was modeled according to the parameters published by Allnér, Nilsson, and Villa.^68^ The structure of filanesib with its pose was directly extracted from the crystal structure (PDB ID 6HKY). The molecule was initially reduced using PyMOL, ensuring that (un)­saturations were correctly reproduced; the aliphatic amine was modeled as protonated, based on its analogy with a lysine side chain. The structure was then optimized to the bioactive minimum using Gaussian16 ^69^ at the B3LYP/6-31+G­(d,p) theory level,^70,71^ followed by a single-point calculation performed at the Hartree-Fock/6–31+G­(d,p) theory level. These calculations served as the starting point to calculate atomic charges using the Merz–Kollman–Singh method.^72^ The output was directly used for parametrization with Antechamber using the GAFF force field^73^ for remaining parameters. The ff14SB force field^74^ was employed to treat all protein residues, while water molecules were modeled according to TIP3P parameters.^75^ Finally, for Na^+^ and Cl^–^, we employed parameters by Joung and Cheatham.^76^

Starting coordinates and topologies for all 4 systems (**ADP**, **ADPfil**, **apo**, and **ATP**) are provided electronically.

### 2.3 Molecular Dynamics Simulations

Molecular dynamics simulations were performed using Amber20.^77^ The 4 systems (**ADP**, **ADPfil**, **apo**, **ATP**) were first minimized by Steepest Descent and Conjugate Gradient methods, initially for 300 steps with positional restraints on all atoms except hydrogen and water and then another 300 steps without restrictions. Solvent equilibration was performed under an NVT ensemble for 9 ps, and the system was gradually heated from 25 to 300 K using the Langevin thermostat over a 20 ps interval. Starting from this stage (and for subsequent stages), 6 independent replicas were initiated for each of the four systems (atomic velocities assigned from different random seeds).

Following heating, each replica entailed equilibration under NpT conditions through a multistage protocol with progressively decreasing positional restraints on C_α_ atoms, first over two short 20 ps simulations, using SHAKE^78^ constraints on bonds containing hydrogen, and Langevin thermostat while maintaining positional restraints on C_α_ atoms, two longer runs, each 1 ns in length, were conducted without restraints. Finally, each of the 6 replicas initiated for each system underwent a 1 μs production stage in the NpT ensemble.

Individual production trajectories resulting from each of the 6 replicas were ultimately concatenated for each system to form metatrajectories that were used for Distance Fluctuation and Shortest Path analyses. The Sander utility was used for the early preproduction stages, while GPU-accelerated pmemd.cuda was used for the later equilibration and production stages. The input files are provided as SI.

A time step of 2 fs was applied to all stages beyond minimization (except for heating, which features a shorter time step of 1 fs). The standard Amber cutoff of 8 Å was applied for the calculation of Lennard-Jones and Coulomb interactions in direct space. Beyond this cutoff, only Coulomb interactions were calculated in reciprocal space. Input scripts for all MD minimization, preproduction, and production stages are provided electronically.

### 2.4 Allosteric Analysis

To analyze and compare allostery across the various systems, we applied Distance Fluctuation Analysis^79^ (DF), and we employed the Shortest Path Map web server^80^ (SPM); codes for both analyses are publicly available (cf. Data and Software Availability). Both techniques have been successfully applied to decrypt allosteric information from unbiased molecular dynamics simulations, both separately and jointly (see, for example, refs 80–82 and references therein).

Distance Fluctuation analysis can be used as a metric of how coordinated two residues in a protein move through an equilibrium MD trajectory or metatrajectory (no fitting or realignment is required; only stripping to leave Cα atoms). The method generates a single *N* × *N* matrix (where *N* is the total number of residues). The individual DF scores *DF*_*ij*_ correspond to a measure of the allosteric coupling between the *i*th and the *j*th residue, given by eq 1

1 $$DF_{ij}=\langle {\left(d_{ij}-\left\langle d_{ij}\right\rangle \right)}^{2}\rangle$$

where *d*_*ij*_ is the distance between the C_α_ of two residues (*i*, *j*) in a given frame, and ⟨*d*_*ij*_⟩ is the average distance between their C_α_s over the whole trajectory. The lower a *DF*_*ij*_ score is, the more allosterically coordinated (or rather allosteric) a residue pair *ij* is. Moreover, since in this work we mainly focus on allosteric differences in coordination among states of the cycle, we employed Δ*DF*, which is simply the difference between *DF* matrices of a final state and an initial state.

On the other hand, SPM overlays on the protein structure a map of the main path through which the bulk of allosteric communication takes place. The method is explained in more detail in the relevant publication by Osuna,^81^ but as a general explanation, it initially relies on a measure of how (anti)­correlated, on average, every residue pair *ij* is during the MD simulation. This value *C*_*ij*_ (from a *N* × *N* correlation matrix **C**) is calculated for each pair according to eq 2

2 $$C_{ij}=\frac{\left\langle \Delta r_{i}\cdot \Delta r_{j}\right\rangle }{\sqrt{\left\langle \Delta r_{i}^{2}\rangle \langle \Delta r_{j}^{2}\right\rangle }}$$

where Δ*r*_*i*_ and Δ*r*_*j*_ are the relative displacements of the C_α_ of the residues *i* and *j* with respect to their position in the most representative structure, averaged along the trajectory (⟨⟩).

Each residue *i* becomes a node on a node-and-edge graph and will be connected by an edge to another node/residue *j* only if their C_α_s remain, on average, closer than 6 Å during the MD simulation, as quantified from an average distance matrix **d**. The length *l*_*ij*_ of each edge is calculated as per eq 3

3 $$l_{ij}=-\log\left(\left|C_{ij}\right|\right)$$

Once the node-and-edge graph is established, an algorithm traces the shortest path to travel from each residue to every other residue. At the end of the process, nodes and edges that are most frequently traveled through (with the threshold chosen arbitrarily) are used to produce a final SPM.

Scripts to calculate correlation and distance matrices **C** and **d** required to perform SPM are provided electronically, as are the scripts to preprocess metatrajectories prior to their calculation. Indeed, the calculation of **C** and **d** for each system first requires prior derivation of an average structure from that system’s metatrajectory, after aligning all frames on C_α_ atoms to eliminate rotational and translational degrees of freedom. An RMSD-based clustering procedure is then conducted on C_α_ atoms of the realigned metatrajectory, and the centroid of the most populated cluster is finally used as the reference structure to calculate **C** and **d**.

### 2.5 Clustering

To analyze and monitor different possible conformations of L5, a separate RMSD-based clustering procedure with *cpptraj* ^83^ was adopted. In the first step, every trajectory was stripped of its side chains and aligned to the backbone of its first frame. In the second step, after alignment of the secondary structures (selection based on those residues that were classified as stable secondary structures in **ATP**, **ADP**, **apo**, and **ADPfil** alike), an averaged structure was calculated. In the third step, trajectories were fitted to a subselection of α2 helix (residues 111–116 and 135–140) of the averaged structure, and only the backbone of residues 111–140 (α2 helix and L5) was retained. In the last step, an RMSD-based hierarchical agglomerative clustering was performed, with *ε* = 5 Å. This procedure was repeated separately for **ATP**, **ADP**, **apo**, and **ADPfil**, obtaining different clustering results; input scripts and output clusters are provided electronically.

## 3 Results and Discussion

### 3.1 General Results for Distance Fluctuation (DF) Analysis

Since we were trying to explain the differences among the different catalytic states, we thought that a more eloquent answer would be achieved by plotting the DF differences after every catalytic event (Figure 2). This was done by subtracting, from the DF values of every catalytic state, those of the previous state (e.g., the changes produced by the ATP hydrolysis are represented by subtracting the DF matrix of **ADP** from **ATP**). Since the state that filanesib binds to is **ADP**, the effect of filanesib binding was depicted by subtracting the **ADP** values from the **ADPfil** DF values. The differences resulting from this operation were plotted in difference matrices, which we call Δ*DF*. Positive values of Δ*DF* (colored in red) indicate a loss of coordination and, therefore, a loss in allosteric connection. Negative values of Δ*DF* (colored black) represent an increased coordination between residues after the (un)­binding/catalytic event. Intermediate values are depicted in white, and they represent regions with similar coordination in both states. The original DF matrices from which these Δ*DF* matrices were calculated can be found in the Supporting Information (Figure S1).

SWI and SWII domains behave as expected, since these two domains are sensitive to the presence or absence of the nucleotide.^15,47,54^ Also, the two switch domains contain key residues in the catalysis of ATP hydrolysis, so a slightly higher coordination is expected in **ATP** compared to **ADP**. When ATP binds to the motor domain (Figure 2B), an increase in the coordination of both regions is observed. ATP hydrolysis and phosphate release produce a mild decrease in the coordination of these regions with the rest of the protein. The most pronounced case is found when the nucleotide is released from the motor domain, which produces the highest loss of coordination of SWI and SWII with the rest of the protein. The binding of filanesib causes the loss of coordination of SWII, while for SWI, the coordination increases moderately. DF values (Figure S1) indicate that L5 presents the lowest coordination in **ADP**.

This variation of coordination is clear when taken together with the Δ*DF* values. L5 appears to have some implication in the binding to the MT, since its allosteric coordination increases when kinesin-5 rebinds to it (at the moment of ADP release), and it remains coordinated at the moment of ATP binding. The binding of filanesib appears to increase the allosteric coordination of L5, but we attributed this to the stabilizing effects that any ligand has on the surrounding residues when bound. The implications of L5 for kinesin processivity will be further discussed in the following sections.

Strong and consistent variations in DF are observed in the N-terminal segment (residues 1–16) for all transitions. A common feature in all forms is the low coordination of this region (Figure S1), which forms an α-helix in all simulations. It has been suggested that the N*-*terminus of motor domains of kinesins behaves as an intrinsically disordered region (IDR) that plays a role in kinesin processivity,^23,84^ with some examples found in the literature for instances of nonhuman kinesin-5.^85–87^ This is consistent with our simulations, since conventional force fields tend to compact IDRs into organized structures.^88,89^ Another region of interest is α0 (residues 30–36), which appears to be highly sensitive to the presence of the nucleotide. For instance, at the moment of ATP binding, it displays a lower Δ*DF* score with L8, α4, and α6, whereas at the moment of ADP release, it shows a generalized increase in DF score with the rest of the protein (Figure 2B and D). α0 could be a key domain for kinesin processivity due to its proximity to helix α6, which directly connects to the neck linker and the nucleotide-binding pocket.

The last domain that presents notable differences across the catalytic states is L8 (residues 166–206), whose sequence is rich in polar and charged amino acids such as serine, lysine, arginine, glutamate, and aspartate. The coordination of L8 appears to increase significantly at the moment of ATP binding. ATP hydrolysis causes a loss of coordination, but the greatest increase in DF score is found when ADP is released (Figure 2D). Additionally, the binding of filanesib causes a decrease in DF scores in the last segment of L8 (residues 186–193). In 2022, a computational study by Guo et al. demonstrated that salt bridges contributed more than hydrogen bonds to the binding of kinesin-5 to the MT, and that these ionic interactions were most abundant with β-tubulin. Moreover, they identified that the three residues that most contribute to these interactions are Glu166, Arg181, and Lys197,^55^ all three of which are located in L8. They also proposed that the kinesin directionality could be a consequence of the preferential interaction of the positively charged surface of kinesin-5 with the negatively charged surface of β-tubulin. Although the stepping process of kinesin-5 might probably be a combination of the passive diffusion of the unbound motor head and a “pull” from the forward head at the moment of nucleotide binding (which causes the docking of the neck linker), it could also include an initial “L8 binding phase”. In this way, a flexible L8 could be the first region to contact tubulin through ionic interactions, which are less affected by distance and directionality than by other molecular interactions.

This would explain why **apo** presents a higher DF score for L8 than **ATP**, even though they both bind to the MT. Once this first step is completed, L8 would contribute to orient the motor domain to fulfill the rest of interactions; this is plausible since the middle region of L8 is highly coordinated with the rest of the protein, as the low Δ*DF* values suggest. The end of L8 directly connects allosterically with the α3 helix, which is part of the binding site of filanesib. As mentioned above, this last segment of L8 has a decreased Δ*DF* score in **ADPfil** compared to the others. This could be interpreted as a rigidification of L8 upon filanesib binding, which negatively impacts the ability of kinesin-5 to bind MT. This is supported by the recent findings of Alexandar and Ulaganathan, who found that the MT binding enhanced the **ATP** motor domain flexibility while also increasing its coordinated movement.^57^

### 3.2 General Results for SPM

DF results do not discriminate involvement of the motor domain core (which is formed by 8 β-sheets) across catalytic stages, as they show indiscriminately low |Δ*DF*| scores in all cases, suggesting a generally high degree of internal allosteric coupling. In this respect, SPM results are extremely useful. These agree with the DF results with respect to the allosteric relevance of the β-sheet core but, in addition, they also show the connections across the different domains (Figure 3). SPMs of all catalytic forms display a “central stem” that diagonally crosses the β-sheet core, from β_2_-sheet to β_4_-sheet, which are in direct contact with L2 and L8, respectively, (both part of the MT interface). In response to the presence of the nucleotide, the branch patterns of the central SPM stem change: **ATP** has the most branched stem (i.e., the most articulate allosteric communication pathway), followed by **apo** and finally **ADP** and **ADPfil**. In **ATP,** there is an allosteric pathway that connects SWI and SWII with the β-sheet core that is present only in this form, as expected from the relationship between these two response elements and the γ-phosphate of ATP; this doubtlessly has an impact on the allosteric behavior of the motor domain.

**Figure 3:**
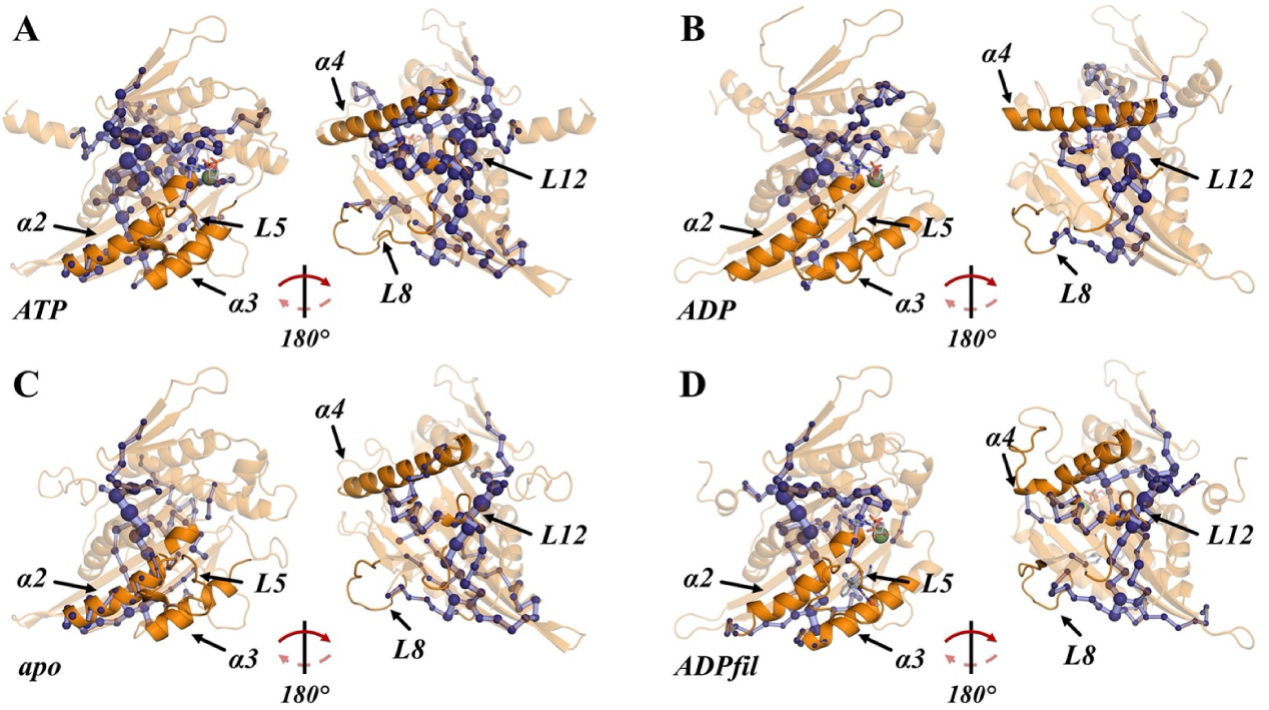
Shortest path maps (SPMs) for **ATP** (A), **ADP** (B), **apo** (C), and **ADPfil** (D) forms. The nodes are represented by dark blue spheres, while paths are represented by a light blue color.

The ATP form also has a highly correlated branch that connects the β-sheet core to L12 and the α4-helix. This branch is also present to a lesser extent, both in length and significance, in **apo** and **ADP**, while **ADPfil** does not present this branch at all. In addition, in **ATP** and **apo** (but not in **ADP** and **ADPfil**), the β-sheet core branches in a way that includes the whole β_4_-sheet, which is in direct contact with L8. L12 and the α4-helix are also part of the MT interface, but, as opposed to L2 and L8, they interact with the α-tubulin monomer. A second difference is that this interaction is governed by hydrogen bonds, while, as mentioned earlier, the interaction with β-tubulin is electrostatic.^55^ This kind of weaker interaction (hydrogen bonds) is also required since the kinesin needs to be able to both attach to and detach from the MT.

The question is, since both **ATP** and **apo** strongly bind to the MT, why is this allosteric connection more intense in the former than in the latter? The most logical explanation is that L2 and L8 are responsible for the “strong binding” component of the kinesin to the MT (ionic bonds), while L12 and α4-helix produce, at the moment of the binding of ATP and due to the establishment of new hydrogen bonds, a finer tuning for the binding of the motor domain. Since hydrogen bonding is characterized by a strong dependency on distance and orientation, it does make sense that they are not present at the initial stages of the binding, i.e., the apo form. Nonetheless, this first approximation of the motor domain to the tubulin dimer in the MT generates a more favorable situation for hydrogen bond formation and, therefore, an “ATP-induced fitting”. This “ATP-induced fitting” is supported by experimental observations in other kinesins that the angle between the β-sheet core and the MT axis varies depending on the presence of the nucleotide (apo or AMPPNP-bound), in a “twisting” movement;^31^ this rotation of the motor domain could complement the docking of the neck linker in propelling the back head toward its front.

Another difference among the catalytic states involves L8. We have already mentioned that the presence of filanesib induces a decrease in the DF score (increase in allosteric communication) of the final segment of L8. From the SPM results, we observe that there is an allosteric path in all forms that directly connects the N*-*terminus of the α3-helix with L8. This path varies depending on the catalytic state, being the largest in length and intensity for **ADPfil**, followed by **ADP,** and finally **apo** and **ATP** (both show a shorter path). All of this supports the hypothesis that L8 needs flexibility to properly bind the MT (**apo** and **ATP**) while its allosteric rigidification achieves the opposite effect (**ADP** and **ADPfil**). Since helix α3 connects both SWI and L8, it is very likely that it mediates the response of L8 to the presence or absence of the γ-phosphate. This is, however, paradoxical since this would make **ADP** and **apo** behave alike, as both lack this phosphate, so the behavior of L8 must be also controlled by its interface with the β_4_-sheet, as mentioned earlier. Lastly, it should be noted that there is a path that connects L5 with the P-loop through helix α2 in **ADP**, **ADPfil,** and **ATP**, this connection being more intense in the two latter (see Section 3.4).

In summary, the allosteric communications among the different regions of the motor domain are mediated through the β-sheet core, and L8 is more allosterically connected in the low MT-affinity states.

### 3.3 Formation of the L5 Binding Pocket

A characteristic feature of the kinesin-5 L5 binding pocket is that it only seems to exist in the presence of its ligands, as this pocket is not detected in ligand-free, ATP- or ADP-bound crystallographic structures. This is in contrast to other allosteric pockets (including the allosteric pocket framed by helices α4 and α6), of which at least a small part is detectable in the absence of ligands. As mentioned earlier, kinetic experiments support the idea that the binding of L5 inhibitors takes place at either the ATP- or ADP-form. It is thus accepted that this binding site is induced by the presence of the inhibitors.^90^ Our DF results support this proposal, as shown by the increase in Δ*DF* scores for the residues that form L5 with respect to the rest of the protein; this can be interpreted as an increase in the loop conformational flexibility required by the inhibitors to fully develop the allosteric binding site. In **ADPfil**, there is a drop in Δ*DF* values that resembles **apo** and **ATP**, but this is only due to the presence of filanesib, which acts as a nexus between L5 and the rest of the protein, since ligands tend to stabilize (or coordinate the movement of) the surrounding residues. These specific variations in DF scores suggest that L5 conformational interconversions as kinesin-5 loops through catalytic states have an impact on its function. This hypothesis finds an experimental validation in the fluorescence assays carried out by Cochran and Gilbert in 2005, who presented a model of an “open/closed” L5,^91^ and Muretta and coworkers, who proposed the alternation of 3 conformational states of L5 in the different catalytic stages; furthermore, they reported that the L5 conformation corresponding to the ADP state shows the highest mobility among the three.^25^ To further test these findings, we performed a clustering of L5 conformations across our MD simulations of all catalytic forms (Figure 4). We found that the most representative conformations in **ATP** and **apo** forms were identical, and, strikingly, we found that it was the same conformation present in **ADPfil**. On the other hand, L5 was found to be significantly more flexible in **ADP**, alternating between 3 main conformational clusters, only one of which resembles that observed in the presence of filanesib (Figure 4C).

**Figure 4:**
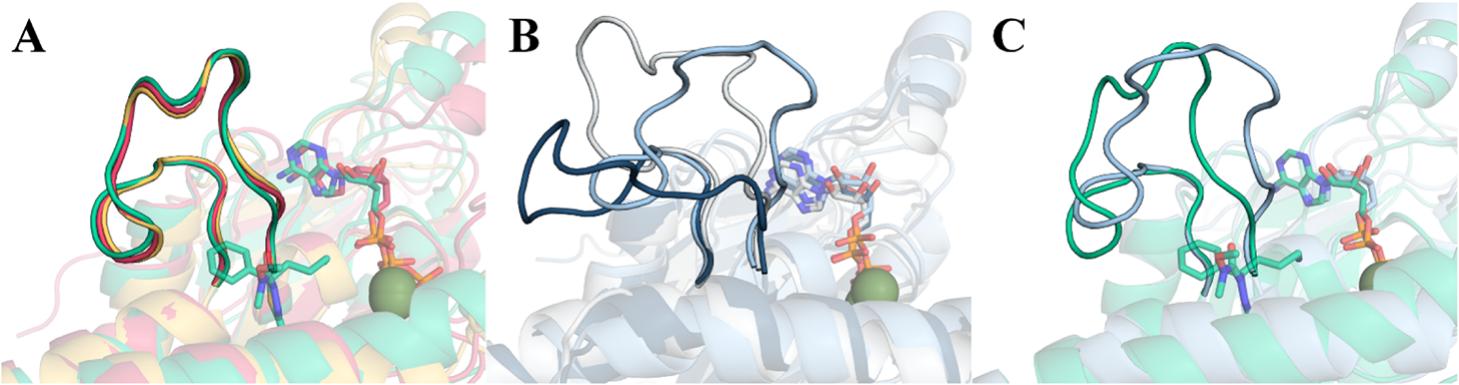
Clustering results of the L5 conformations in all simulated states. A) Superposition of L5 in **ATP** (red), **ADPfil** (green), and **apo** (yellow). B) Most representative conformations of L5 in **ADP**. The dark blue conformation is the most populated one (63%), followed by the cyan conformation (24%) and the white one (13%). C) Superposition of L5 conformation in **ADPfil** (green) and the most similar conformation in **ADP** (cyan).

In a similar way to the behavior of the neck-linker, which alternates between “docked” and “undocked” conformations during the catalytic cycle, we propose that L5 also alternates between a “docked” conformation in **ATP** and **apo** (with strong binding to MT) and an “undocked” conformation in **ADP** (with weak binding to MT). In the case of **ADPfil**, L5 appears to be locked into a conformation that remarkably resembles the “docked” conformation present in **ATP** and **apo** (Figure 4a). Curiously, clustering results also show that L5 conformations in **ADP** that could allow binding of filanesib come from scarcely representative clusters (Figure 4b, cyan and white conformations), somewhat supporting the idea that the L5 binding pocket is tendentially cryptic and prone to opening in the presence of inhibitors.

### 3.4 Loop 5 Is Allosterically Connected to the ATP Binding Site

An interesting feature of L5 inhibitors is that, while they lock the L5 loop into apo- and ATP-like conformations as just discussed, they generally stabilize the kinesin motor domain in an ADP-like conformation (rather than inducing apparent deformations in the entire kinesin motor domain). Experimental studies show that the L5 inhibitors indeed slow the release of ADP and thereby reduce the kinesin affinity for the MT.^37^ In addition, previous molecular dynamics studies on the motor domain show that L5 inhibitors increase the binding affinity of ADP to its binding site.^46,54^ In the publication by Behnke-Parks et al.,^92^ it is suggested that the effect of these inhibitors could be transmitted to the SWI domain through the α3 helix. We have not seen any elements that suggest that connection. However, in our SPM study, we could detect a strong allosteric pathway that directly connects L5 with the nucleotide binding site (P-loop) present in **ATP** and **ADPfil** (Figure 5). A similar connection can be detected in **ADP**, but it is not as strong as that in the former two. In **apo**, however, this path is not present (Figure 5). In **ATP** and **ADPfil** simulations, the SPM starting from Gly115 (L5 loop) reached all the way up to the P-loop, being the most intense in the former. The neighboring L5 residue, Glu116, was found to be critical for the kinesin function in mutational studies^93^ and engages in an ionic bond with the primary amine of filanesib and many other kinesin-5 inhibitors,^52^ so this interaction should be taken into account in the rational design of inhibitors. In 2013, McGrath and coworkers showed that Thr107 in the P-loop was key to the catalytic hydrolysis of ATP due to the interaction with Glu270 (SWI), which, in turn, is also the anionic component of the ionic bridge that exists between SWI and SWII (Arg240-Glu270).^47^

**Figure 5:**
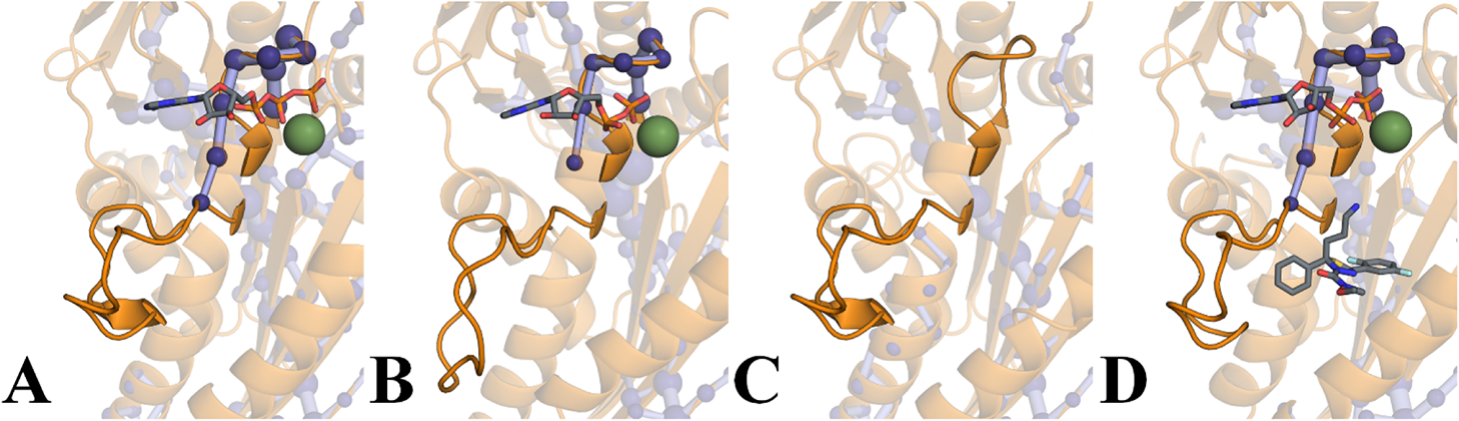
SPM connection between L5 (Glu116-Gly134) and P-loop (Gly105-Thr112) in **ATP** (A), **ADP** (B), **apo** (C), and **ADPfil** (D).

Both our SPM and DF studies show that L5 motion is also coupled to that of SWI in **ADP**, **ATP,** and **ADPfil**. As mentioned in the Introduction, L5 differences among the entire kinesin superfamily suggest its role in finely modulating the kinetics of the motor domain. Our results support this idea, since L5 is allosterically connected to both the nucleotide-binding site and the MT interface.

### 3.5 Trp127 Is Crucial for the Functional Interaction between L5 and Helix α3

In several previous studies, it has been noted that Trp127 in L5 is displaced by inhibitors, in some cases becoming available for inhibitor stabilization through π-stacking interactions.^39,46^ Also, an inspection of the available crystal structure for kinesin-5 bound to the ATP analogue AMPPNP (PDB ID: 3HKY^62^) reveals a possible π–π stacking interaction between Trp127 and Tyr211 on helix α3, an interaction that is not present in the ADP form. More interesting insights are given through mutational studies, in which mutations Trp127Ala and Trp127Cys reduced the catalytic activity of kinesin-5, this being attributed to a hindered release of ADP.^25,26^ Moreover, this was partially reversed when the fluorescent probe monobromobimane (mBBr) was attached to the cysteine mutant,^25^ which suggests that mBBr, with a similar size and structure to the indole system, could mimic the presumed π–π interactions of Trp127. The possibility that Tyr211 acts as a “π–π anchoring point” between L5 and helix α3 is especially attractive since Tyr211 is in the part of the α3 helix that connects to SWI; in this way, L5 conformations could be indirectly influenced by the presence/absence of the γ-phosphate. Our DF results agree with these experimental observations. A deeper analysis of the region corresponding to L5 in **ATP** and **apo** trajectories reveals that DF values are not homogeneous within the loop, being particularly low for Trp127 and increasing on either side as one moves away from Trp127. This, together with the fact that there is no other apparent interaction of L5 residues with helix α3, further suggests that Trp127 is the anchoring residue of L5 (Figure 6). Even immediately adjacent residue (Glu128), which could also engage with helix α3 through electrostatic interaction with Lys207 from α3, presents a higher DF score. Quantitative measures of the π–π interaction of Trp127 and Tyr211 and of the ionic interaction between Glu128 and Lys207 are provided in the Supporting Information (Figures S2 and S3, respectively). The importance of the role of Trp127 is additionally reinforced by evolutionary preservation of L5−α3 interactions akin to Trp127–Tyr211. An example is found in the kinesin-5 of *Plasmodium falciparum* PfK5 (PDB ID 7NB8 ^94^) in which two tyrosines (Tyr244 and Tyr245) are found in a similar position as Tyr211 in α3 of the human kinesin-5. An analogous situation can be found in the yeast kinesin-5 from *Schizosaccharomyces pombe* (Cut7) in which a salt bridge between Asp208 (L5) and Arg298 (α3) appears to stabilize L5 in a docked position in the crystal structure (PDB ID: 6S8M^95^). This means that targeting this kind of interaction could be a plausible strategy for inhibiting kinesin-5 from other species for which there is no known L5 inhibitor. More in general, sequential conservation of Trp in analogous positions to Trp127, and an aromatic residue (Tyr or Phe) in analogous positions to Tyr211, is deducible from the multiple sequence alignment of kinesin-5 homologues in selected species that we present as Figure S4.

**Figure 6:**
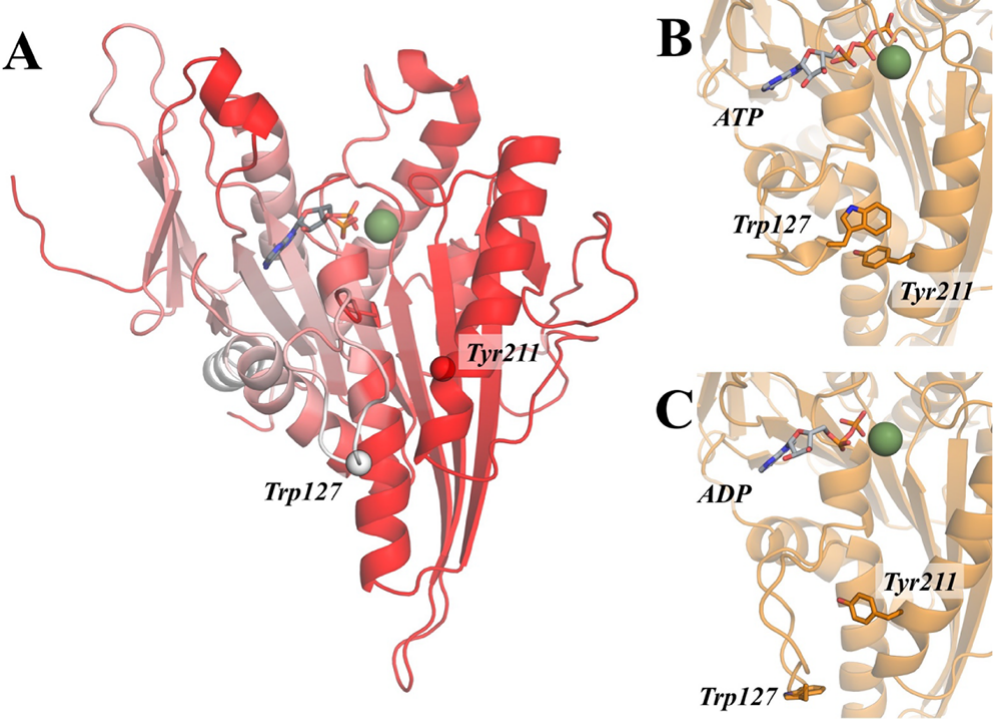
A) Projection of Δ*DF*_127,*j*_ scores (**ADP** - **ATP**) for Trp127. The red color follows the same metrics as the matrix in Figure 2C and indicates a decrease in coordination of Trp127 with the rest of the protein. B–C) Crystal structures of **ATP** (B) and **ADP** (C). Trp127 and Tyr211 are shown as sticks. In **ATP**, both residues have an orientation that suggests a π–π interaction, while in **ADP** this is not observed.^56,58^

### 3.6 Allosteric Inhibitors Conformationally Lock L5, Increase the ADP Binding Affinity, and Hamper MT Binding

As mentioned earlier, filanesib and other L5 inhibitors are experimentally recognized to lock the motor domain in kinesin-5 in a low MT-affinity state and slow the ADP release. Intuition would suggest that this is a direct consequence of trapping the catalytic cycle in the ADP state, since filanesib would prevent the docking of L5 by blocking the interaction of Trp127 with Tyr211 on helix α3, and this interaction is necessary for a fully active kinesin. However, our quantitative analysis shows that filanesib actually favors the π–π stacking interaction between those residues (Figure S2), and our clustering analysis indicates that filanesib stabilizes the loop in the same conformation as in **apo** and **ATP**. Also, from Figure S2, it is clear that the previously reported^61^ loss of the Trp127–Tyr211 interaction in **ADPfil** can still occur, but only occasionally.

An additional proof that this cannot be the mechanism of inhibition is provided by Tcherniuk and coworkers, who showed that the mutation Trp127Ala confers only moderate resistance to *S*-trityl-*L*-cysteine (STLC), possibly due to the loss of π–π interactions with the inhibitor, but not to monastrol.^96^ If interference with the interaction between Trp127 and Tyr211 were part of the mechanism of action of L5 inhibitors, then mutations of Trp127 should definitely have an impact on monastrol or STLC efficacy.

From previously reported MD studies, it is also known that the presence of L5 inhibitors increases the binding affinity of ADP.^46,54^ In the absence of the inhibitor, the binding of ATP is stronger than that of ADP, which agrees with the natural course of events, since the kinesin in the apo form should avidly capture ATP while, once hydrolyzed, it should allow nucleotide release.^54^ Apart from the connection that exists between L5 and the nucleotide-binding site, which is mediated by helix α2 (see Section 3.4), we hypothesized the existence of a direct interaction of L5 with the nucleotide. This idea is not new and was previously proposed by Harrington and coworkers in 2011, who suggested that the length of L5 conferred it a large diversity of conformations, some of which bent onto the nucleotide pocket; but, to achieve those conformations, they had to run simulations at high temperature (600 K).^97^ This means that there is a simpler explanation for the higher binding affinities detected in the MD simulations. Upon inspection of the crystal structure of ATP-bound kinesin-5, we detected a possible interaction of Glu118 on L5 with the ribose ring of the nucleotide. We hypothesized that this interaction could be more significant in **ATP** and **ADPfil**, which would explain the higher binding affinity of the nucleotide in those states.

However, a quantitative analysis (Figure S5) revealed that the H-bond between Glu118 and nucleotide is less present when the loop is fixed in a “docked” conformation (**apo**, **ATP,** and **ADPfil**). Instead, this bond is more frequent in **ADP**. These results rule out such a hypothesis. A more detailed analysis suggests that the role of Glu118 is the opposite. To ensure progression of the catalytic cycle, **ADP** must leave the nucleotide binding site. In this way, an interaction with Glu118, which is directed toward the solvent-exposed region of the nucleotide binding site, would help the ADP “escape” from the kinesin. However, when filanesib binds to the protein, it pushes the nucleotide back into its ATP-like form by disrupting the H-bond interaction between Glu118 and ADP. Intrigued by what could be disrupting this interaction and rendering **ADPfil** more like **ATP**, we renewed our focus on Glu118 in **ADPfil** and saw that it is actually the NH_3_^+^ group of filanesib that essentially sequesters Glu118 through the formation of a stable salt bridge: this is clear from the radial distribution function plotted in Figure S5, panel E. This interpretation of the role of Glu118 is supported by yet another analysis on the distribution of the nucleotide density inside the pocket (Figure S6): in **ADP**, the nucleotide seems to be shifted to the exterior of the protein when compared to **ATP**; instead, in **ADPfil**, where Glu118 is sequestered by filanesib, the ADP returns to an ATP-like position (this is supported also by a better superposition between the two densities). As a further indication of the importance of Glu118, we note that in the previously introduced multiple sequence alignment (Figure S4), we observe some degree of sequential conservation of a Glu or Asp at this position.

This “external aid” (provided by Glu118), together with a lower binding affinity of ADP for the protein, would ensure the release of the nucleotide and the progression of the catalytic cycle. This aligns with the kinetic experiments performed by Waitzman and coworkers, who showed that L5 accelerated the release of ADP during the MT binding event. Moreover, they showed that a deletion in loop 5 (residues 126–132) produced a slower release of ADP, and that STLC (an L5 inhibitor) produced the same effect on ADP release.^24^ It is worth mentioning that the release of ADP is stimulated by the presence of MTs, although it can also occur in their absence (as in our simulations); however, it has been experimentally shown that L5 inhibitors such as ispinesib slow the release of ADP even with respect to its reduced rate of release in the absence of MTs.^37^

The likely result of these two observations on the role of Glu118 and the position of ADP in the binding site would be slower mitosis, which is, of course, itself enough to explain the antimitotic effect, since a generalist explanation of the effect of any antimitotic drug is that a long enough mitotic arrest causes the cell to either enter apoptosis or abruptly exit mitosis with the wrong distribution of the genetic material that causes cell death. However, this does not explain the characteristic monopolar phenotype produced by kinesin-5 inhibitors, which is caused by the collapse of the highly tensioned metaphase cellular structure.^38^ As stated in the Introduction, not only does kinesin-5 generate the force that drives the building-up of the mitotic spindle but it is also responsible for upholding the structure. In 2017, Chen and coworkers showed that the collapse of the structure induced by L5 inhibitors was a consequence of kinesins being locked in a low MT affinity state and therefore diffusing away from them.^98^ Not only have our SPM results shown that filanesib is able to keep the allosteric connection present in **ADP** at L8, but they have also shown it is enhanced. This would ultimately explain the phenotypic observations. The simplest answer would be that this is mediated through the α3 helix as a consequence of the insertion of the difluorophenyl moiety of filanesib into the α3 helix. However, our SPM results did not detect any path connecting this pocket with L8. Indeed, this connection cannot be responsible for the low affinity state for the MT, since monastrol and other L5 inhibitors, which do not occupy that pocket also produce such a phenotype. We observe, however, that the final segment of the α2 helix belongs to a path that connects to the β_4_-sheet (interface with L8) in **ATP**, **apo,** and **ADPfil**. This connection might be a consequence of L5 being locked in an ATP-like conformational state, as also patently shown by the supplementary Δ*DF* matrix (**ADPfil – ATP**) reported in Figure S7. We believe that the allosteric connection with L8 in **ADPfil** is the result of L5 being locked in an ATP-like conformation in the presence of a bound ADP that by itself intensifies the allosteric signals to the β_4_-sheet.

To conclude, while it was not within the scope of this work to examine potential allosteric mutation sites in a systematic way, we should comment that the comparison of our combined SPM and DF results for **ADPfil** with those in the other catalytic states should serve as a solid basis to rationalize the effects of any distal mutations known to neutralize the allosteric rewiring introduced by filanesib, as well as to identify any sites where new mutations could develop, provided they are far from the L5 binding site.

## 4 Conclusions

The functionality of kinesin-5 is directly linked to the normal progression of the catalytic cycle within each of its motor domains. The presence or absence of the nucleotide causes reorganization of the allosteric signals that connect the key components of each motor domain. In this work, we have conducted microsecond-long atomistic molecular dynamics simulations of the kinesin-5 motor domain. The substantial length of our simulations allowed us for the first time to reliably compare allosteric traits of four key kinesin-5 forms: ATP-bound (**ATP**), ADP-bound (**ADP**), and **apo**, as well as an ADP-bound form in the presence of the known inhibitor filanesib (**ADPfil**).

Distance fluctuation (DF) analysis of our simulations shows that SWI and SWII coordinate with the rest of the protein when ATP is bound. Additionally, it was shown that L8 coordination with the rest of the protein varies throughout the catalytic cycle, showing the strongest coordination in **ATP**. Shortest path map (SPM) results suggest that this allosteric connection was mediated through the interface between the β_4_-sheet and L8. The presence of filanesib also induces a strong allosteric connection in L8, although in this case, it appears to be mediated through α3 rather than the β_4_-sheet interface.

L5 is undoubtedly coupled to the functionality of the protein. The length and sampling breadth of our simulations allowed us to perform for the first time a thorough conformational analysis showing that L5 oscillates between two states: “docked”, characterized by a high coordination with the rest of the protein, and “undocked”, which displays the opposite behavior. From the DF results, we have demonstrated that this phenomenon is mediated by the π–π interaction of residues Trp127 and Tyr211, and that this interaction is typical in **apo** and **ATP**, whereas it is lost in **ADP**; however, our simulations confirm that the binding of filanesib (**ADPfil**) is able to rescue this interaction better than previously thought. We even managed to show that filanesib is able to prevent the interaction that normally takes place between Glu118 and ADP. As such, this is the first work that provides an accurate description and explanation of the conformations (and their relative populations) that L5 adopts in the representative states of the motor domain through the catalytic cycle. To reiterate, in **ATP** and **apo,** L5 has a stable “docked” conformation characterized by its interactions with α3. ATP hydrolysis leads to an “undocked” conformation of L5 with a higher conformational flexibility; remarkably, filanesib binding induces L5 to mimic the **ATP** state. Our findings support the idea that the allosteric pocket of L5 has a cryptic behavior and that the binding of the inhibitor (which takes place in the **ADP** state) helps to fully develop the cavity. We have also shown that in **ADP**, the (unsequestered) residue Glu118 from L5 could play a role in helping ADP release, which is a necessary step for the catalytic cycle progression; in accordance with previous kinetic experiments,^24^ this supports the idea that L5 can directly interact with the nucleotide binding pocket to promote nucleotide release, and indeed, the interaction is not present in **ATP**. The SPM results also suggest the existence of an allosteric connection with the nucleotide binding site, which is present in **ATP** and **ADPfil**, and, to a lesser extent, in **ADP**.

Even though it was typically assumed that L5 inhibitors forced the motor domain into an **ADP**-like state, we have found that the presence of the allosteric inhibitor filanesib makes the motor domain exhibit an allosteric behavior that is essentially similar to that of **ATP** in the conformation of L5 and the nucleotide-binding site. On the other hand, it resembles **ADP** in terms of the allosteric behavior of L8. Moreover, it appears to disrupt the catalytic cycle through a triple mechanism. First, it is able to enhance the allosteric connection of L5 with the P-loop (as in **ATP**), which might explain the higher binding affinity of the nucleotide when L5 inhibitors are bound to it.^46,54^ Second, filanesib directly diminishes the interaction of the nucleotide with Glu118 present in **ADP** (even if this is not immediately apparent from the crystal structure alone). The consequence of both is the blocking of ADP release from the motor domain, and therefore the interruption of the catalytic cycle. Third, filanesib is able to amplify an allosteric signal that connects helix α3 with the final part of L8, which is present in **ADP** and, to a lesser extent, in **ATP** and **apo**. This, together with the fact that **ADPfil** is not able to mimic the allosteric signal at the β_4_-sheet of **ATP**, might directly lead to a loss of affinity for the MT. To the best of our knowledge, this is the first rational explanation of how L5 inhibitors lock kinesin-5 in a low MT-affinity state. We should add that such remarkable findings would not have been possible without this first systematic comparison of four different kinesin-5 states *in silico*.

## Supplementary Material





## Data Availability

The code to perform Distance Fluctuation analysis is available on Github (https://github.com/colombolab/Distance-Fluctuation-DF-Analysis.git). The Shortest Path Map web server is publicly accessible (https://spmosuna.com/). Modeller is available for download at https://salilab.org/modeller/. Input scripts for MD simulations, as well as subsequent analyses and clustering, are provided as Supporting Information (*vide supra*).

## Notes

DF matrices, Tyr211–Trp127 interaction, Lys207–Glu128 interaction (RDF), multiple sequence alingment of selected kinesin-5 homologues, Glu118–ADP interaction and Glu118–filanesib interaction, comparison of nucleotide densities within the four simulated systems, Δ*DF* matrix between **ADPfil** and **ATP** (PDF)
Input scripts for Modeller, starting coordinates and topologies for all 4 systems, MD input scripts, clustering and calculation of matrices for SPM, clustering input and output (ZIP)
