# Supplementary material for: Molecular Dynamics Simulations Provide Further Insights into the Allosteric Regulation of the Kinesin‑5 Motor Domain by Loop 5
Source: J Chem Inf Model. 2026 Mar 12;66(7):4101–15. doi: 10.1021/acs.jcim.5c02999 (PMC13080997; doi:10.1021/acs.jcim.5c02999)
Supplement: Supplementary file 1 [file ci5c02999_si_001.pdf]

SUPPORTING INFORMATION

Molecular dynamics simulations provide further insights into the allosteric regulation of the kinesin-5 motor domain by Loop 5

Gabriel Rodríguez-Santos[1], Giorgio Bonollo[2], Cristiano Sciva[2], Giorgio Colombo[2], Concepción Pérez-Melero[1,3,*], Stefano A. Serapian[2,*].

[1] Pharmaceutical Sciences Department. Pharmaceutical Chemistry Unit, University of Salamanca. Biomedical Research Institute of Salamanca (IBSAL). Salamanca, Spain.
[2] Chemistry Department, University of Pavia, Pavia, Italy.
[3] Tropical Diseases Research Centre, University of Salamanca (CIETUS), Salamanca, Spain
* Authors to whom correspondence should be addressed:
conchapm@usal.es; stefanoartin.serapian@unipv.it

Contents

Figure S1. DF results for **ATP**, **ADP**, **apo** and **ADPfil**.
Figure S2. Tyr211-Trp127 interaction.
Figure S3. Radial distribution function of Glu128 oxygens ($O_{\varepsilon 1}$,$O_{\varepsilon 2}$) from Lys207 nitrogen (N_{ζ}).
Figure S4. Multiple Sequence Alignment of selected kinesin-5 homologues.
Figure S5. Glu118 interaction with the nucleotide in every state and with filanesib in **ADPfil**.
Figure S6. Results of the grid command on the nucleotide in every state.
Figure S7. ΔDF matrix between **ADPfil** and **ATP**.
References

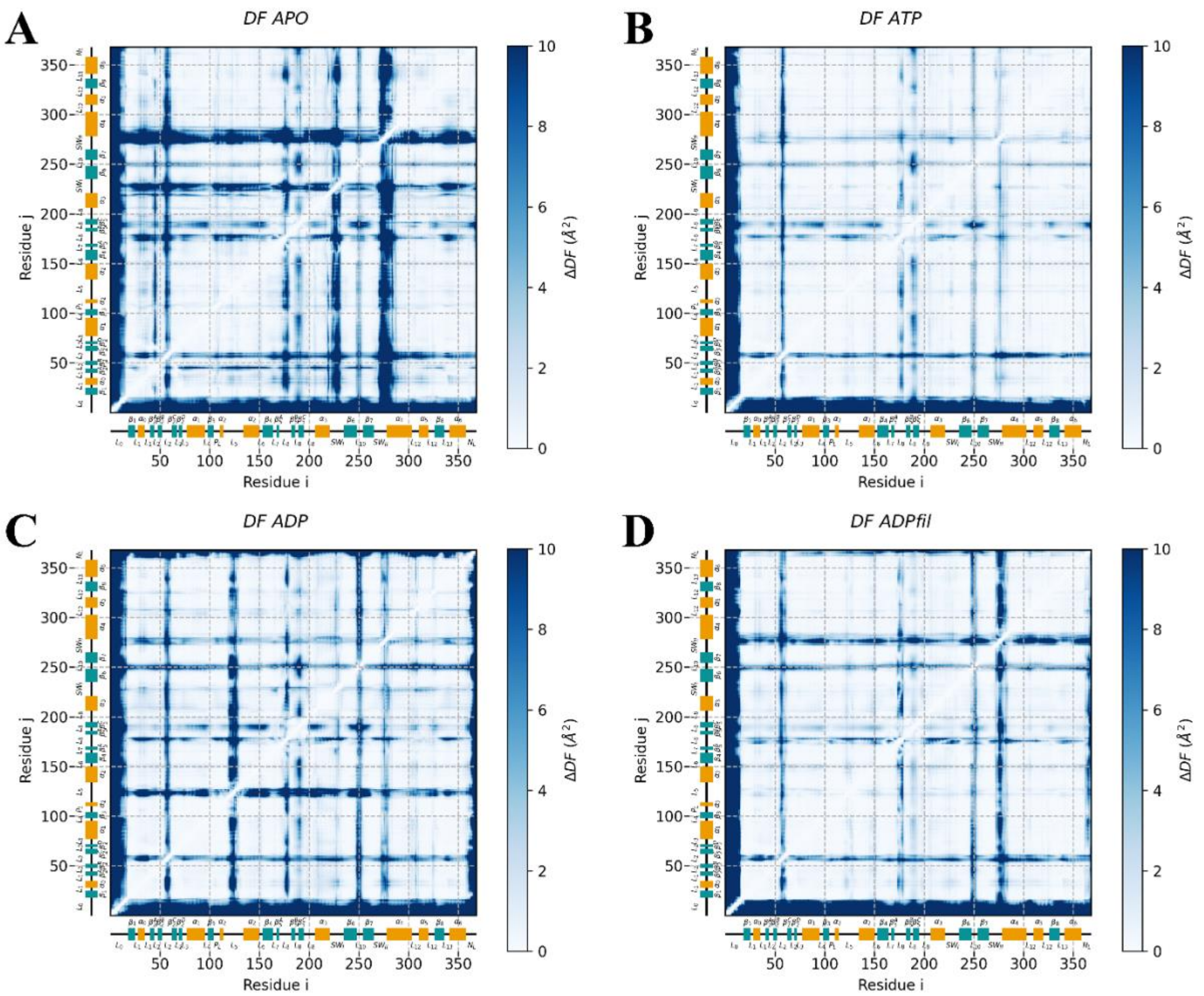

Figure S1. DF results for **ATP** (A), **ADP** (B), **apo** (C) and **ADPfil** (D). A threshold of 10 Å^2 is applied, so that DF values higher than 10 Å^2 are automatically rescaled to 10 Å^2. Intensity of the blue color is correlated to the DF values.

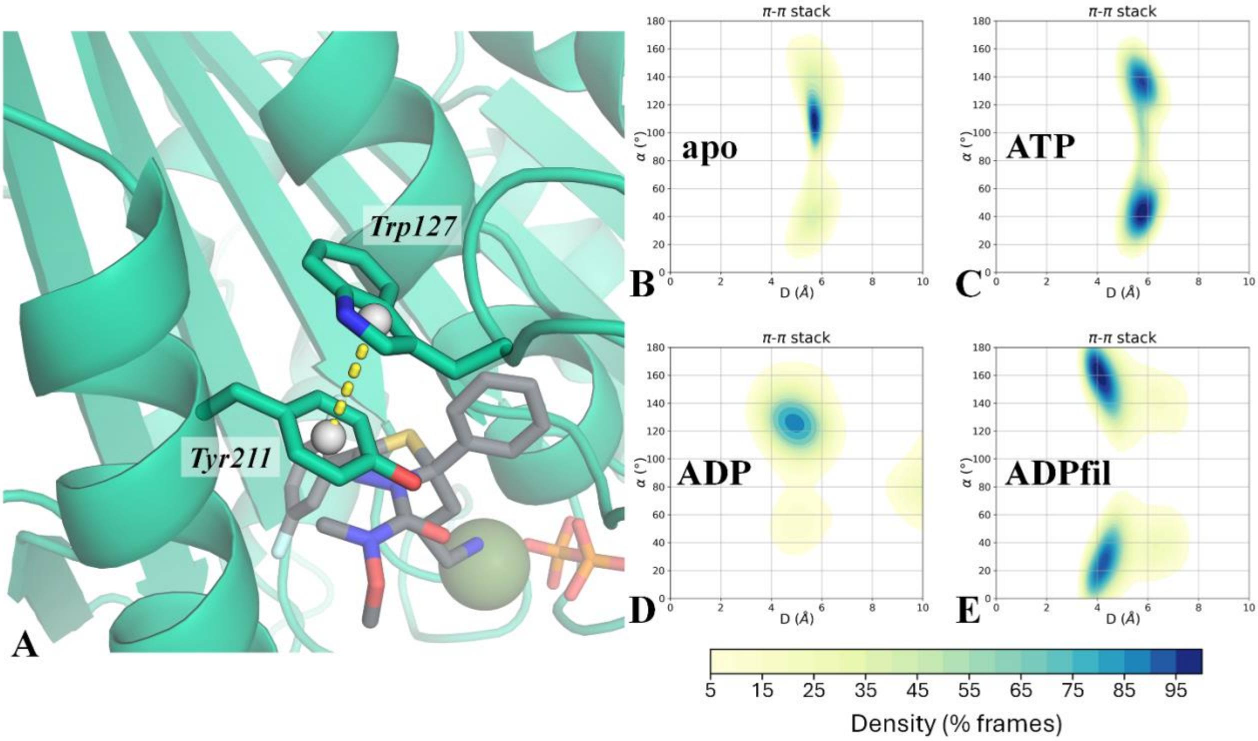

Figure S2. Tyr211-Trp127 interaction. A) Static structure taken from **ADPfil** simulation highlighting the distance between center of mass of the two rings. B-D) Kernel Density Estimate (KDE) plot of the distance between center of mass of the two rings and the angle between the two planes. Only a minor percentage of frames in **ADP** could be involved in $\pi - \pi$ interaction, while in **ATP** and **ADPfil** a stronger interaction is present.

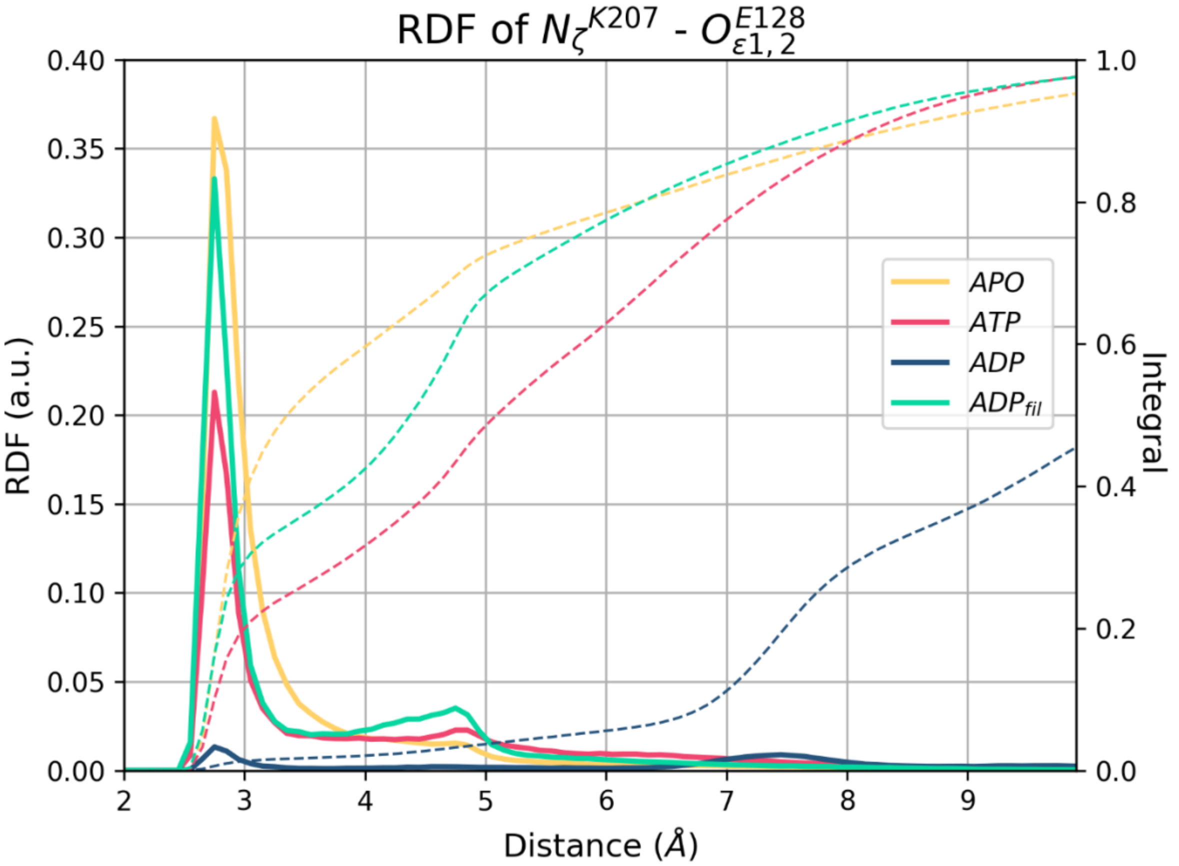

Figure S3. Radial distribution function of Glu128 oxygens ($O_{\varepsilon1}$,$O_{\varepsilon2}$) from Lys207 nitrogen (N_ζ). The RDF values are plotted as solid lines, while dashed lines represent RDF integrals for each system. The peak visible between 2.5 and 3.5 Å highlights the interaction between the two sidechains, which is present only in **apo**, **ATP** and **ADPfil**.

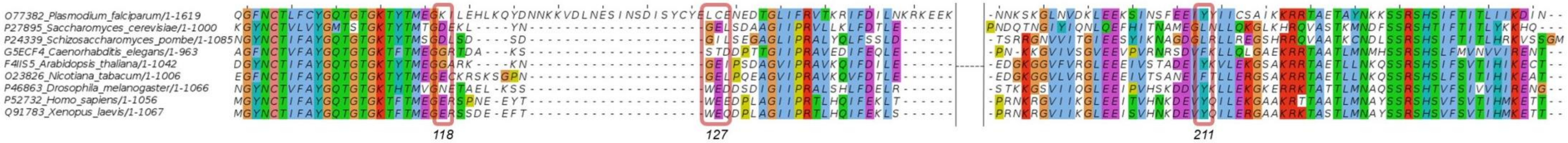

Figure S4. Multiple Sequence Alignment of selected kinesin-5 homologues. On the left we list the Uniprot accession codes of each protein, together with species names and protein length. Sequences were aligned with the Clustal Omega server[1] and results were visualized through Jalview[2].

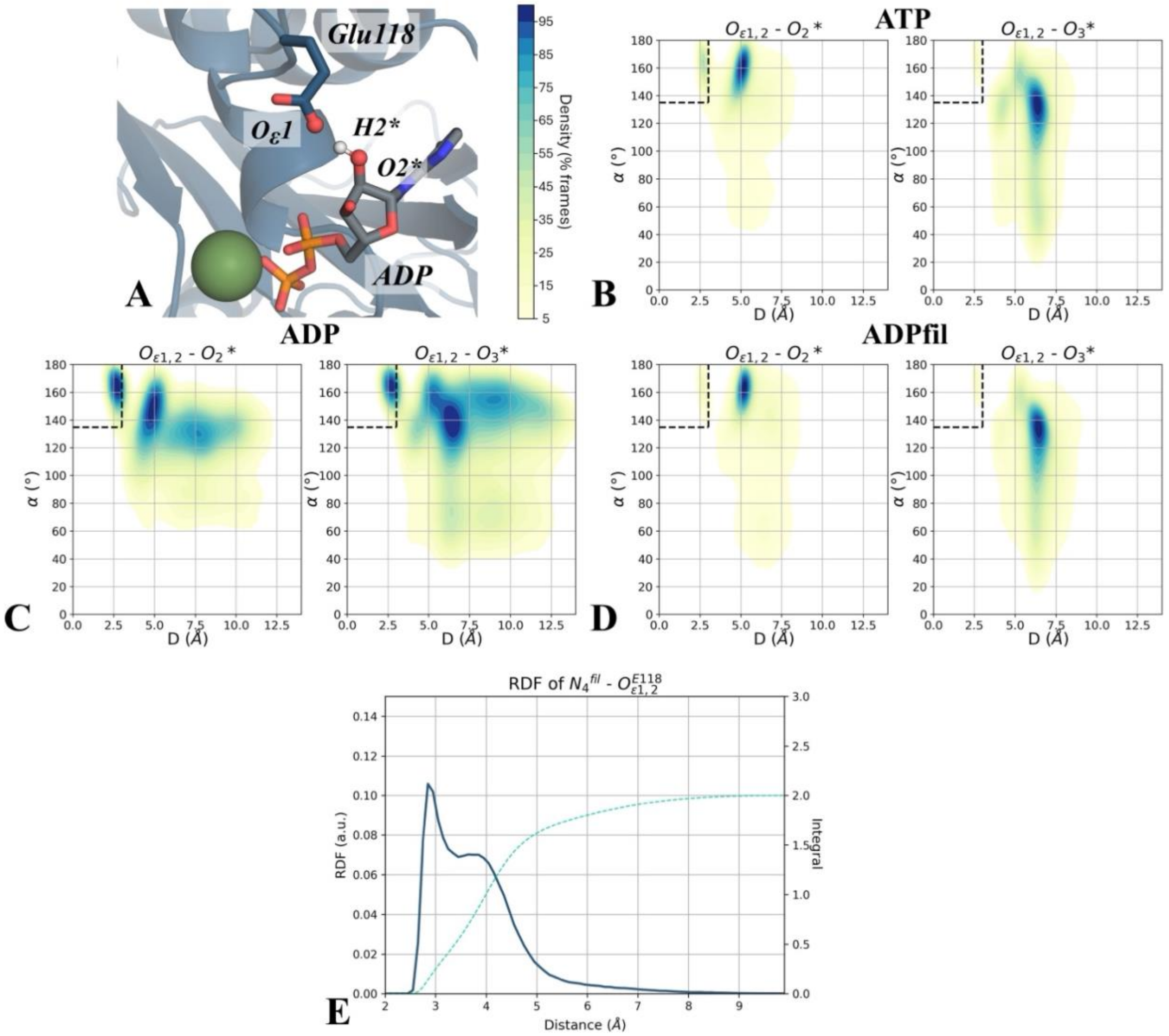

Figure S5. Glu118 interaction with the nucleotide in every state and with filanesib in **ADPfil**. Glu118 interacts more strongly with the nucleotide in **ADP**. A) Static structure taken from **ADP** simulation highlighting the atoms involved in hydrogen bond interactions. B-D) Kernel Density Estimate (KDE) plot of the distance between one oxygen of Glu118 and one of the two oxygens of ADP versus the O-H•••O angle in all the states of the cycle. Only a minor percentage of frames in ATP and **ADPfil** is involved in hydrogen bonds, while in **ADP** a stronger interaction is present. E) The radial distribution function between N_4 in the NH_3^+ group of filanesib and Glu118 oxygens shows, with an intergral of almost 2 within the first 8 Å, that Glu118 is sequestered fairly stably by filanesib.

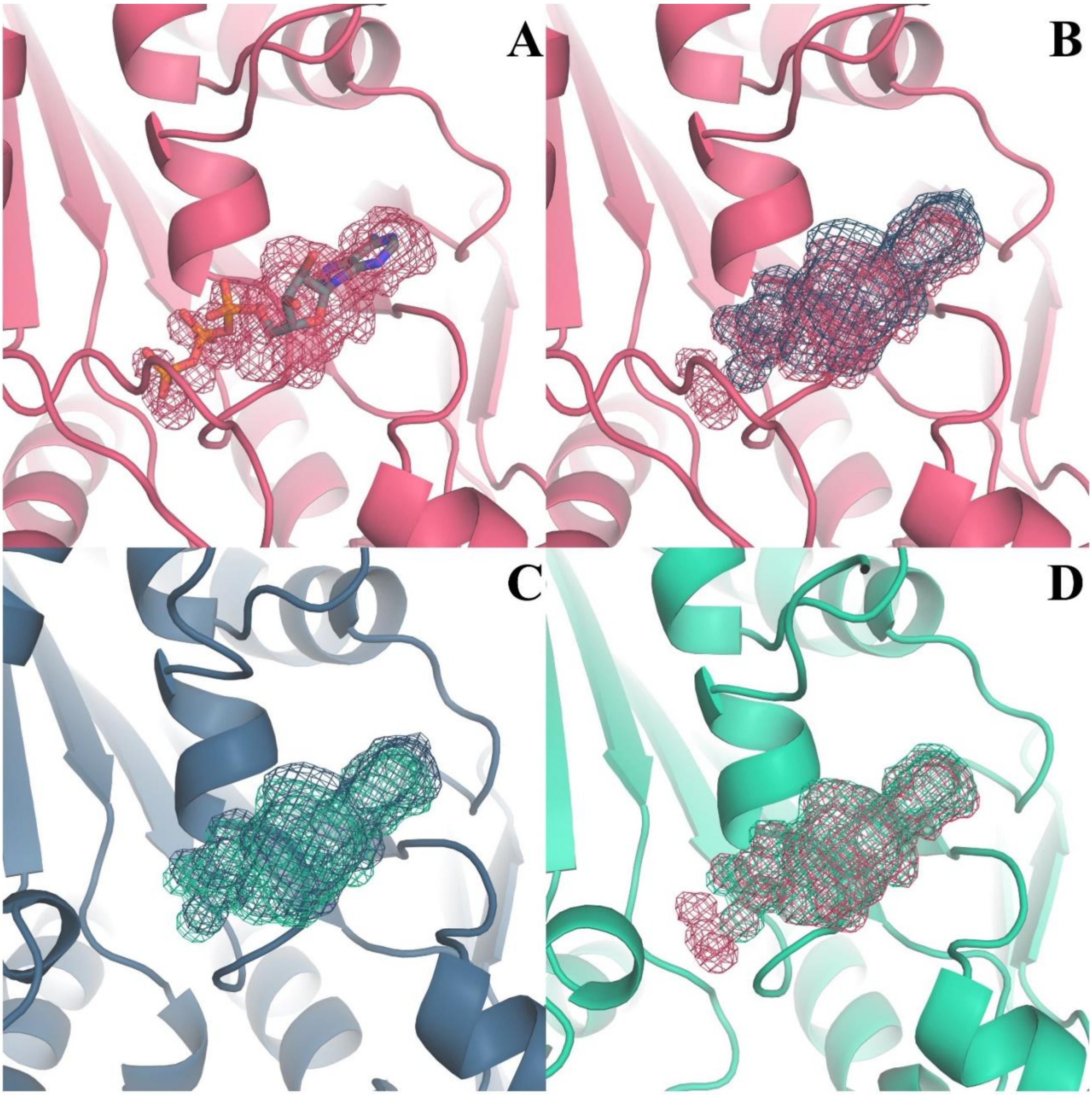

Figure S6. Results of the grid command on the nucleotide in every state. A) 3D density of ATP inside the pocket during simulations of **ATP** superimposed onto a frame of the MD. B) Superposition between the density of ATP in **ATP** (red) and ADP in **ADP** (blue) onto the **ATP** structure; the ADP density seems to be partially shifting towards the upper right part of the pocket. C) Superposition between the density of ADP in **ADP** (blue) and ADP in **ADPfil** (green) on **ADP**; density of ADP in **ADPfil** seems to partially reacquire similarity with ATP conformations. D) Superposition between the density of ADP in **ADPfil** state (green) and ATP in **ATP** (red) on the **ATP** structure; density of ADP in **ADPfil** state seems to match almost entirely the one of ATP in the pocket, supporting the hypothesis that filanesib blocks the **ADP** state in an ATP-like conformation.

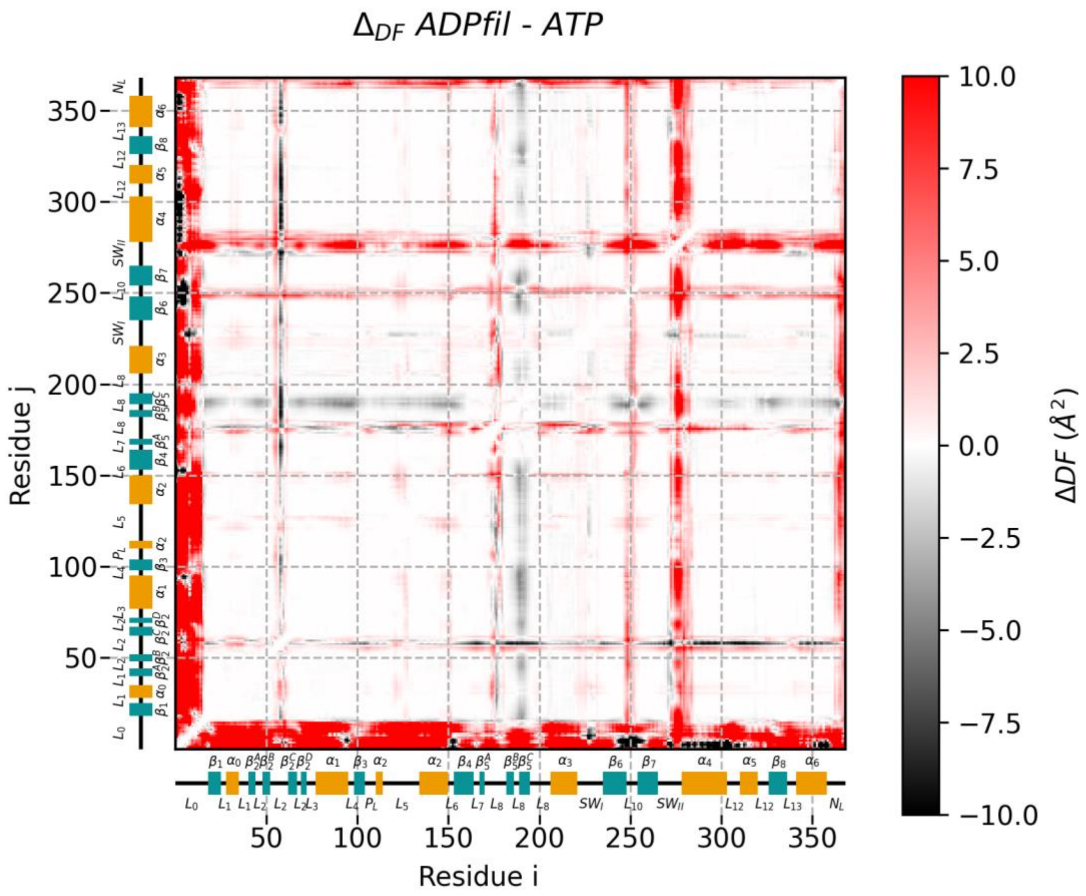

Figure S7. ΔDF matrix between **ADPfil** and **ATP**. With respect to the **ADP-ATP** matrix, the fluctuations on L5 are reduced, painting a picture of the **ADPfil** state as an "ATP-like ADP state", with locked L5. Additionally, it can be seen that the final portion of L8 presents a higher correlation with respect to **ATP.**

References

1. Madeira F, Madhusoodanan N, Lee J, Eusebi A, Niewielska A, Tivey ARN, et al. The EMBL-EBI Job Dispatcher sequence analysis tools framework in 2024. Nucleic Acids Res. 2024;52:521–5.
2. Waterhouse AM, Procter JB, Martin DMA, Clamp M, Barton GJ. Jalview Version 2—a multiple sequence alignment editor and analysis workbench. Bioinformatics. 2009;25:1189–91.
